# Helicobacter Infection and Gastric Adenoma

**DOI:** 10.3390/microorganisms9010108

**Published:** 2021-01-05

**Authors:** Simone Bertz, Miriam Angeloni, Jan Drgac, Christina Falkeis, Corinna Lang-Schwarz, William Sterlacci, Lothar Veits, Arndt Hartmann, Michael Vieth

**Affiliations:** 1 Institute of Pathology, Universitätsklinikum Erlangen, Krankenhausstr. 8-10, 91054 Erlangen, Germany; simone.bertz@uk-erlangen.de (S.B.); miriam.angeloni@uk-erlangen.de (M.A.); william.sterlacci@klinikum-bayreuth.de (W.S.); arndt.hartmann@uk-erlangen.de (A.H.); 2 Institute of Pathology, Klinikum Bayreuth, Preuschwitzerstr. 101, 95445 Bayreuth, Germany; jan.drgac@klinikum-bayreuth.de (J.D.); christina.falkeis@klinikum-bayreuth.de (C.F.); corinna.lang-schwarz@klinikum-bayreuth.de (C.L.-S.); lothar.veits@klinikum-bayreuth.de (L.V.)

**Keywords:** gastric adenoma, intestinal tubular adenoma, foveolar-type adenoma, pyloric gland adenoma, oxyntic gland adenoma, familial adenomatosis coli, gastritis, Helicobacter, autoimmune

## Abstract

Background: We aimed to provide insight into the actual frequencies of gastric adenoma types and their association with gastritis status and associated mucosal changes with a focus on Helicobacter infection and the operative link on gastritis assessment (OLGA)/operative link on gastric intestinal metaplasia assessment (OLGIM) staging. Methods: From the archive of the Institute of Pathology in Bayreuth, we collected a consecutive series of 1058 gastric adenomas diagnosed between 1987 and 2017. Clinicopathological parameters retrieved from diagnostic reports included adenoma type and localization, associated mucosal changes in antrum and corpus (i.e., type of gastritis, the extent of intestinal metaplasia and atrophy), gender, date of birth, and date of diagnosis. Results: Intestinal-type adenoma was the most frequent adenoma (89.1%), followed by foveolar-type adenoma (4.3%), pyloric gland adenoma (3.4%), adenomas associated with hereditary tumor syndromes (2.8%), and oxyntic gland adenoma (0.4%). Adenomas were found in the background of *Helicobacter pylori* (*H. pylori*) gastritis in 23.9%, Ex-*H. pylori* gastritis in 36.0%, autoimmune gastritis in 24.8%, chemical reactive gastritis in 7.4%, and others in 0.1%. More than 70% of patients with gastric adenomas had low-risk stages in OLGA and OLGIM. Conclusions: We found a higher frequency of foveolar-type adenoma than anticipated from the literature. It needs to be questioned whether OLGA/OLGIM staging can be applied to all patients.

## 1. Introduction

According to the current World Health Organization (WHO) classification system [1], gastric dysplasia (syn.: glandular intraepithelial neoplasia low-grade and high-grade) is defined as unequivocal neoplastic changes of the gastric epithelium without evidence of stromal invasion. The two-tiered grading system of gastric dysplasia using the terms low- and high-grade is based on the Padova international [2] and Vienna/revised Vienna classifications [3,4] and was adopted by the current (2019) WHO-classification [1]. Within the WHO classification system, intestinal tubular and pyloric gland adenomas are separated from gastric dysplasia in different chapters [1].

There has been confusion about the term adenoma in the literature. Partly, the term dysplasia has been used for endoscopically flat or depressed lesions and the term adenoma has been used for protruded sessile or pedunculated polypoid lesions with dysplasia [5]. However, according to the current consensus, adenoma is defined as a low-grade dysplasia regardless of its endoscopic appearance, whether sessile, flat, depressed, or pedunculated [1]. According to the Japanese approach regarding the diagnosis of gastric adenoma, adenoma is defined as non-invasive low-grade neoplasia [6], whereas any high-grade intraepithelial neoplasia is classified as intramucosal carcinoma, irrespective of its endoscopic appearance [6], in contrast to the WHO and associated classifications.

Among gastric polyps, there is a reported frequency of gastric adenomas < 1%, with a prevalence increasing with patient age [7]. Geographical differences and incidence are mainly due to its association with Helicobacter infection and eradication therapies [8,9]. For pathologists, the knowledge of the histomorphological spectrum of adenoma is essential since there are several differential diagnoses with non-adenomatous non-neoplastic polypoid lesions in the stomach. These lesions include fundic gland polyps (Elster’s cysts), hyperplastic (hyperplasiogenic) polyps, and reactive or regenerative polypoid changes of the gastric mucosa, e.g., (post-) inflammatory polyps. The distinction between gastric adenoma and non-adenomatous lesions is important since, according to the current European Society of Gastrointestinal Endoscopy (ESGE) guideline update for the management of epithelial precancerous conditions and lesions in the stomach (MAPS II), staging and treatment are recommended not only for endoscopically visible lesions with high-grade dysplasia and carcinoma but also for low-grade lesions [10]. This is due to the strong association with gastric cancer [8]. Progression rates from adenoma to high-grade intraepithelial neoplasia are 15%, and progression rates from adenoma to cancer are up to 59% [11,12,13]. Moreover, with reported frequencies of 18.7%, upgrading from low-grade intraepithelial neoplasia in the biopsy specimen to high-grade intraepithelial neoplasia in the endoscopic resection seems to be common [14]. Adenomas can be associated with somatic mutations but also with hereditary tumor syndromes, most frequently familial adenomatous polyposis (FAP) and its variant Gardner syndrome [9]. The most frequent FAP-associated gastric adenoma type is foveolar-type adenoma (FovA), often at the top of Elster’s cysts [9].

Intestinal-type adenoma (TubA) is the most frequent type of adenoma, it usually occurs in the 6th decade in a background of Helicobacter gastritis. Bleeding is a typical symptom of large lesions and may lead to anemia and hematochezia [15]. Histomorphologically, TubA resembles colorectal tubular adenoma. Compared to gastric type adenomas, the progression of TubA to high-grade dysplasia and adenocarcinoma seems to be much more frequent [16], and combined intestinal and gastric type lesions seem to harbor an even higher risk of progression [17]. Apart from Adenomatous Polyposis Coli (*APC*) mutations, which occur in about 60% of non-invasive dysplastic lesions including adenoma and high-grade disease, but rarely in gastric carcinoma, molecular alterations in TubA overlap with those of gastric cancer: microsatellite instability (MSI) has been reported in 27% [15].

FovA is a rare lesion, which occurs in an otherwise healthy gastric body and fundic mucosa most frequently in the context of FAP syndrome, associated with alterations of the *APC* gene, and rarely as sporadic lesions [15,16]. Histomorphologically, there is no difference between sporadic and syndromic lesions. Both present with columnar foveolar-type epithelium with stratified nuclei and characteristic apical Periodic Acid-Schiff (PAS)-positive mucin caps [18]. Syndromic FovAs frequently occur simultaneously on top of fundic gland polyps, sometimes also with pyloric gland adenomas [19]. Some authors stated controversially that foveolar-type dysplasia is more aggressive than intestinal-type dysplasia [17]. In fact, FovA is a lesion with a low risk of progression [18].

Pyloric gland adenoma (PGA) accounts for about 2.7% of gastric polyps and occurs most frequently in females in the 7th decade [20]. It is most frequently localized in the gastric body and fundus [20] but can also arise from pyloric metaplasia or gastric heterotopia in the pancreato-biliary system and other sites [21,22]. PGA consists of pyloric glands with small or cystically dilated, closely arranged tubuli and cuboidal epithelium with pale, slightly eosinophilic cytoplasm and round, inconspicuous nucleoli [20]. On the molecular level, *GNAS* and *KRAS* mutations are characteristic and frequent events in PGA [23]. Moreover, PGA is a genetically unstable lesion with chromosomal alterations comparable to gastric adenocarcinoma [24]. MSI has been found in PGAs in Lynch syndrome patients, but not in sporadic cases [25]. Foci of gastric type adenocarcinoma have been reported in up to 47%; therefore, complete excision of PGA is required [26]. Syndromic PGA has been described in FAP patients, juvenile polyposis, Lynch syndrome, and others [19,25,27].

Oxyntic gland adenoma (OGA) is a rare lesion, usually encountered in patients between 60 and 70 years [28]. This mostly polypoid lesion is composed of oxyntic and chief cells arranged in variable patterns [29,30]. Risk factors are not well known, but acid-suppressive medication has been discussed, whereas inflammation and associated mucosal changes have not yet been brought into context [28,29,31]. OGA and gastric adenocarcinoma of the fundic gland type are morphologically similar lesions and some authors believe that they are the same lesion [30]. The majority of OGA cases is limited to the mucosa. For cases with submucosal spread and atypical features, diagnosis of carcinoma is suggested due to the potentially aggressive clinical behavior in those cases [28]. Until now, no recurrences or metastatic lesions have been reported [28,29].

In this study, we present a large consecutive cohort of 1058 gastric adenomas diagnosed between 1987 and 2017 at the Institute of Pathology in Bayreuth. Data were retrieved from diagnostic reports and the relationship between adenoma types and their respective mucosal environment was analyzed, with a focus on Helicobacter infection. Pointing at the consecutive character of this study, the most important aim of this work was to provide insight into the actual frequencies of adenoma types and their association with gastritis types and associated mucosal changes.

## 2. Materials and Methods

The study population consisted of 1058 consecutive cases with a diagnosis of gastric adenoma retrieved from the records of the Institute of Pathology, Bayreuth, Germany. In order to maintain the character of a consecutive cohort, we excluded consult cases from this analysis.

The initial classification of adenoma samples, which was based on ten groups (i.e., TubA, PGA, FovA, OGA, mixed PGA/FovA, multiple TubA, mixed PGA/TubA, TubA in FAP, FovA in FAP, and TubA in Gardner syndrome), was reduced to five groups (i.e., TubA, PGA, FovA, OGA, and syndromic) according to the following criteria:

- Three cases with mixed adenomas, two with combined PGA and FovA and one with PGA combined with TubA, were grouped together and classified as PGA since PGA is the prognostically relevant lesion [20];
- Seven cases with multiple TubAs were grouped together with singular TubAs;
- Patients with FAP (twenty-four TubAs and four FovAs) and Gardner syndrome (two TubAs) were grouped together and classified as Syndromic.

Patients sharing the same date of birth (117/1058) were considered as having recurrent or multiple lesions and were not excluded from the cohort. Variables used for clinicopathological analysis included age and gender as well as adenoma localization within the following compartments of the stomach: antrum, corpus, intermediate zone, cardia, and “remaining stomach”. The latter term was used to denote the compartment of the stomach whenever no exact location was provided in the clinical information. Adenoma type and concurrent pathologies of the gastric mucosa were retrieved from pathology reports and documented separately for antrum and corpus. These pathologies included gastritis types (i.e., *Helicobacter pylori* (*H. pylori*), corpus dominant *H. pylori*, Ex-*H. pylori* after prior successful eradication therapy, chemical reactive, autoimmune, Crohn’s and lymphocytic) and grading of the associated mucosal changes (i.e., intestinal metaplasia and atrophy). Starting from data on gastritis types a new variable was established, summarizing antrum and corpus gastritis diagnoses by combining them according to the following criteria:

- We classified cases with any active *H. pylori* gastritis in antrum and/or corpus and corpus dominant *H. pylori* gastritis as “*H. pylori* gastritis”. In this group, we also included lymphocytic gastritis as it is frequently associated with active *H. pylori* gastritis [32] and as there were no patients with celiac disease;
- Cases with atrophic autoimmune gastritis (A-gastritis) in the corpus and chemical reactive gastritis (C-gastritis) in the antrum were classified as “A-gastritis”;
- Cases without any documented inflammation in the antrum and corpus were classified as “no gastritis”;
- “Ex-*H. pylori* gastritis” was used to classify cases with chronic, non-active gastritis.

In addition, data on intestinal metaplasia and atrophy were used for the operative link on gastritis assessment (OLGA) [33] and for the operative link on gastric intestinal metaplasia assessment (OLGIM) [34] classifications (Stages 0–4) according to the four-tiered Sydney system (no, mild, moderate, severe) [35].

All analyses were performed relying on the R statistical framework v. 4.0.3 (R Core Team (2020). R: A language and environment for statistical computing. R Foundation for Statistical Computing, Vienna, Austria, URL https://www.R-project.org/). Associations between categorical variables were calculated relying on Pearson Chi-square test, whereas the non-parametric Wilcoxon test for independent samples was used to analyze quantitative variables. When evaluating the association between adenoma type (or gastritis type) and IM/atrophy, two different 2-level classification-systems were used: in the first one, OLGIM and OLGA stages 2, 3, and 4 were collapsed into a unique class re-named “stage >= 1”, and in the second one, stages 0–2 were collapsed into a “low-risk” group and stages 3–4 were collapsed into a “high-risk” group, as proposed in the literature [36]. All statistical analyses involving antrum and corpus gastritis were performed after removing the only Crohn’s gastritis sample (1/1058). *p*-values < 0.01 were considered statistically significant.

The study was approved by the Ethics Committee of the Friedrich-Alexander University, Erlangen-Nürnberg and is part of the OMG study on gastritis status and neoplasia, reference 145-15Bc.

## 3. Results

### 3.1. Clinicopathological Characteristics of the Cohort

The clinicopathological characteristics of the cohort are summarized in Table 1. All adenoma samples were classified into five groups as explained in detail in Materials and Methods: mixed adenomas were classified according to the prognostically relevant lesion (PGA); cases with multiple adenomas were merged in the TubA and all adenomas associated with hereditary tumor syndromes were grouped together as syndromic adenomas.

### 3.2. Development of Adenoma Diagnosis with Time

**Figure 1:**
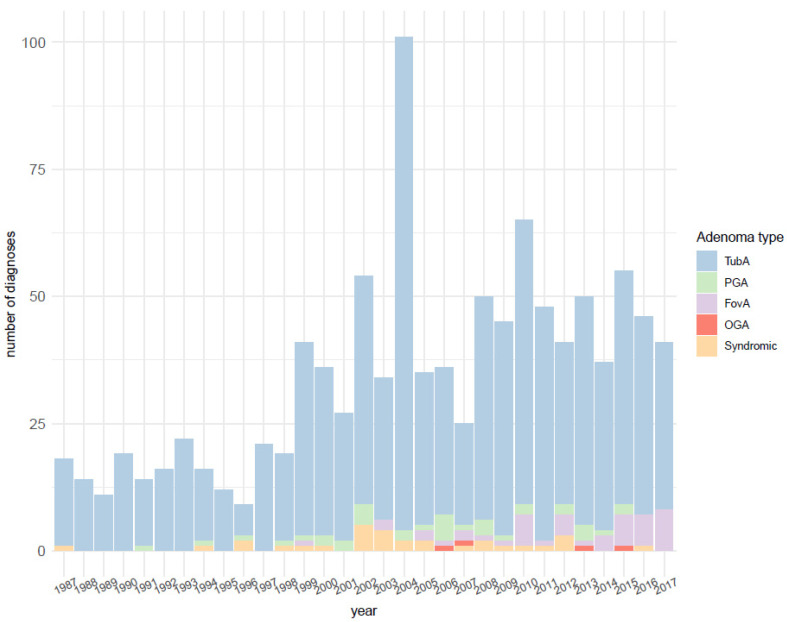
Distribution of the number of adenoma diagnoses over the investigated time frame (1987–2017) at the Institute of Pathology, Bayreuth. TubA: intestinal-type adenoma; PGA: pyloric gland adenoma; FovA: foveolar-type adenoma; OGA: Oxyntic gland adenoma.

A total of 1058 gastric adenoma samples diagnosed between 1987 and 2017 were retrieved. An increase in the number of diagnoses over the investigated time frame, peaking in 2004, can be detected. TubA appears to be the most diagnosed adenoma type throughout the whole period of time. There are only very few diagnoses of gastric type adenomas before 2000 and afterward, and the number of diagnoses remains constantly below 10 per year. Figure 1 depicts the development of adenoma diagnosis with time at the Institute of Pathology, Bayreuth.

### 3.3. Distribution of Adenoma Types by Gender and Age

**Figure 2:**
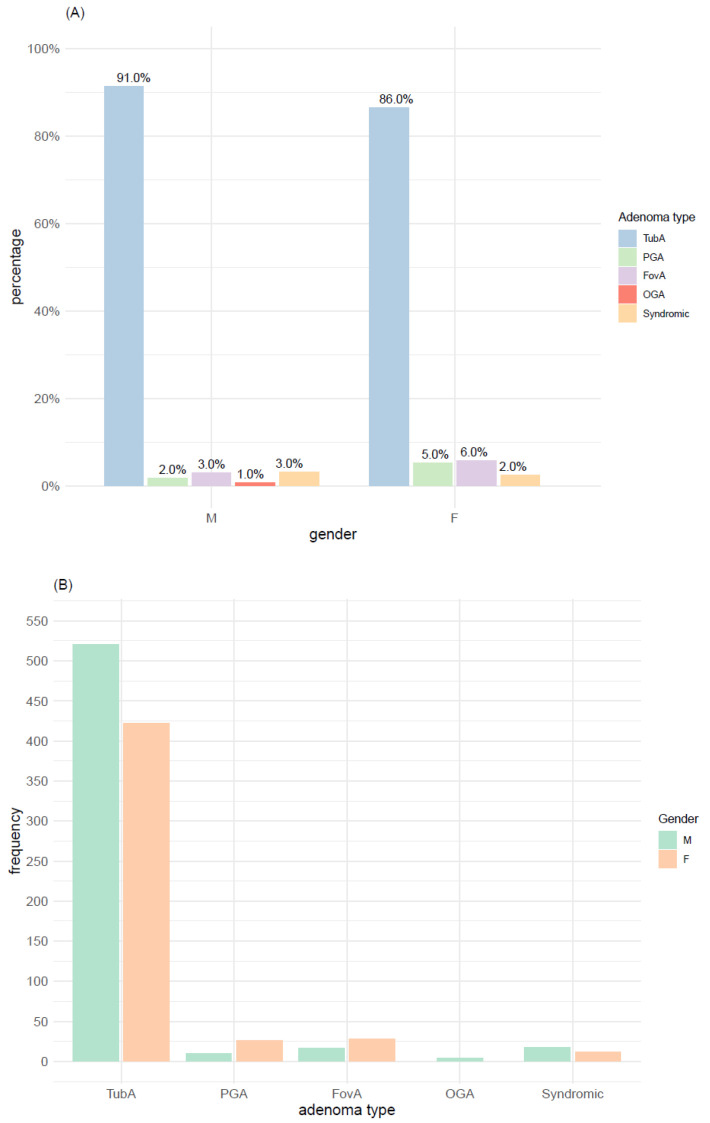
Adenoma type and gender. (**A**) Adenoma type distribution within gender. (**B**) Gender distribution within adenoma types. TubA: intestinal-type adenoma; PGA: pyloric gland adenoma; FovA: foveolar-type adenoma; OGA: Oxyntic gland adenoma; M: male; F: female.

TubA accounted for 90% and 86% of adenoma types in males and females, respectively (Figure 2A). Gender distribution within each adenoma type (Figure 2B) was analyzed relying on one-proportion Z-test. A significantly higher proportion of females compared to males had PGA (*p*-value = 0.003), whereas no significant difference in the proportion of males and females was detected for the remaining adenoma types (*p*-value > 0.01).

**Figure 3:**
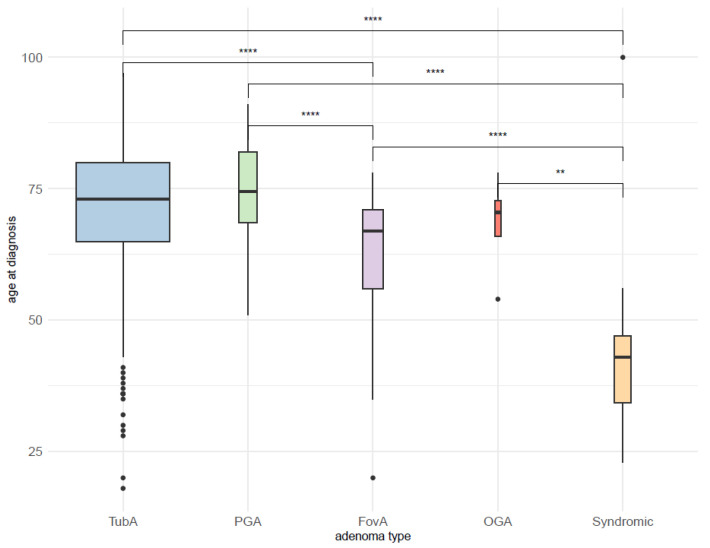
Distribution of age at diagnosis for each adenoma type. ****: significant difference with *p*-value < 0.0001; **: significant difference with *p*-value < 0.01; TubA: intestinal-type adenoma; PGA: pyloric gland adenoma; FovA: foveolar-type adenoma; OGA: oxyntic gland adenoma.

In the studied cohort, the age at diagnosis ranged from 18 to 100 years (mean = 70.1 years, standard deviation (SD) = 12.6 years, interquartile range (IQR) = 64–79 years), with almost all syndromic cases except one in the lower quarter. The distribution of age at diagnosis for each adenoma type is shown in Table 2. According to the Wilcoxon test, the median age at diagnosis of syndromic adenomas was significantly lower than the median age of the remaining adenoma types. A significant difference in age distribution was also found between FovA and TubA as well as between FovA and PGA (Figure 3).

### 3.4. Distribution of Gastric Adenoma Types by Localization

**Figure 4:**
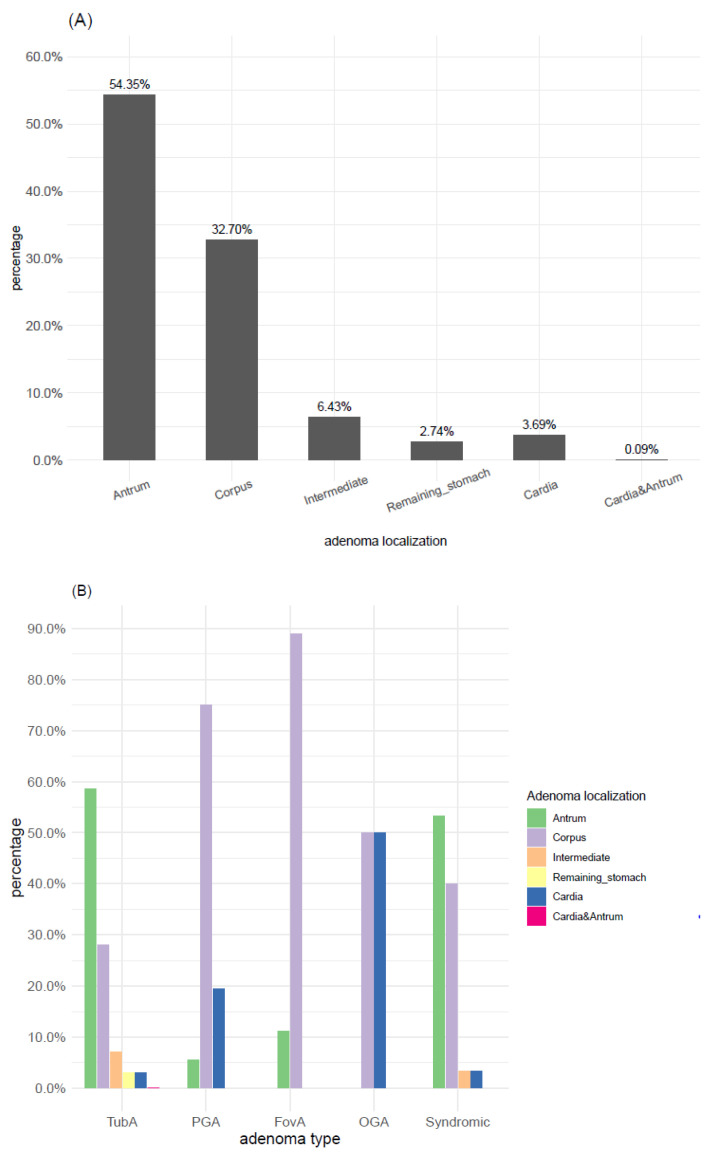
Adenoma type and localization. (**A**) Frequency distribution of adenoma localization. (**B**) Distribution of adenoma localization within adenoma types. TubA: intestinal-type adenoma; PGA: pyloric gland adenoma; FovA: foveolar-type adenoma; OGA: oxyntic gland adenoma.

The majority of adenomas (93.48%) were localized in the gastric antrum and corpus, including the intermediate zone (Figure 4A). Almost 60% of TubAs were localized in the antrum, whereas the corpus turned out to be the predilection site for PGA (75%) and FovA (88.9%). The four OGA samples were instead equally distributed between corpus (50%) and cardia (50%) (Figure 4B).

### 3.5. Association of Gastric Adenoma Types with Gastritis

Gastritis was documented in 999/1058 (94.4%) cases, whereas for the remaining cases gastritis status was not documented in the report since no additional biopsies from antrum and corpus were taken. Thus, in almost 6% of all cases, guidelines were not obeyed.

The most frequent type of gastritis was Ex-*H. pylori* gastritis (36.0%) after successful eradication therapy, followed by A-gastritis (24.8%), *H. pylori* gastritis (23.9%), and C-gastritis (7.4%). No gastritis was reported in 7.8% of cases, whereas the only case of Crohn’s gastritis (0.1%) was removed from further statistical analyses.

**Figure 5:**
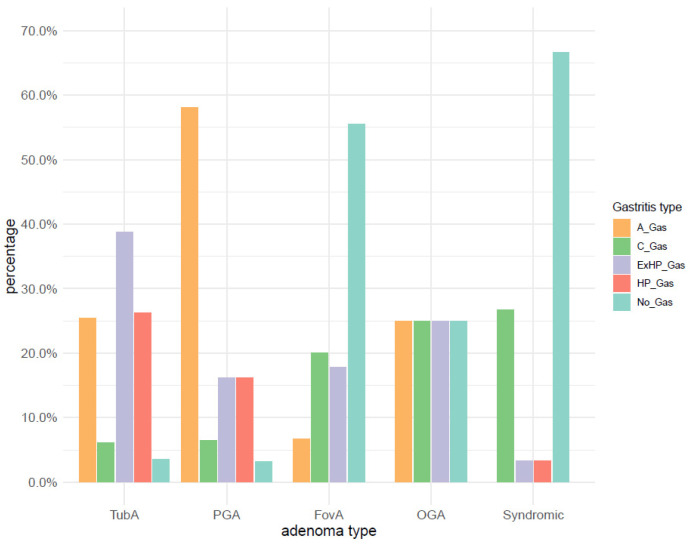
Distribution of the main gastritis groups within adenoma types. TubA: intestinal-type adenoma; PGA: pyloric gland adenoma; FovA: foveolar-type adenoma; OGA: oxyntic gland adenoma; A_Gas: autoimmune gastritis; C_Gas: chemical reactive gastritis; ExHP_Gas: Ex-*H. pylori* Gastritis; HP_Gas: *H. pylori* Gastritis; No_Gas: no gastritis.

The distribution of the main gastritis groups (as defined in the Materials and Methods section) within adenoma types (Figure 5) indicates that TubA occurs mainly in the background of *H. pylori*, Ex-*H. pylori*, or A-gastritis, accounting for a total of 90% of all TubA cases. The majority of foveolar and syndromic adenomas occurred in pristine antrum mucosa (55.6% and 66.7%, respectively), whereas 58.1% of pyloric gland adenomas were associated with A-gastritis. No predominant gastritis type was detected for the four OGA samples.

93.3% (28/30) of syndromic adenomas occurred in uninflamed mucosa, one occurred in the context of *H. pylori* gastritis, and one in the context of Ex-*H. pylori* gastritis. These findings suggest that there is almost no syndromic adenoma with a background of active inflammation.

Looking at special types of gastritis before grouping them for statistical analysis, there were 20 cases with Corpus dominant *H. pylori* gastritis and five cases with lymphocytic gastritis of the corpus mucosa. Among the lymphocytic gastritis cases, two were combined with active *H. pylori* gastritis in the antrum and one with Ex-*H. pylori* gastritis. Ninety percent (18/20) of Corpus dominant *H. pylori* gastritis cases were found in the background of TubA, whereas the remaining 10% (2/20) were found in PGA.

**Figure 6:**
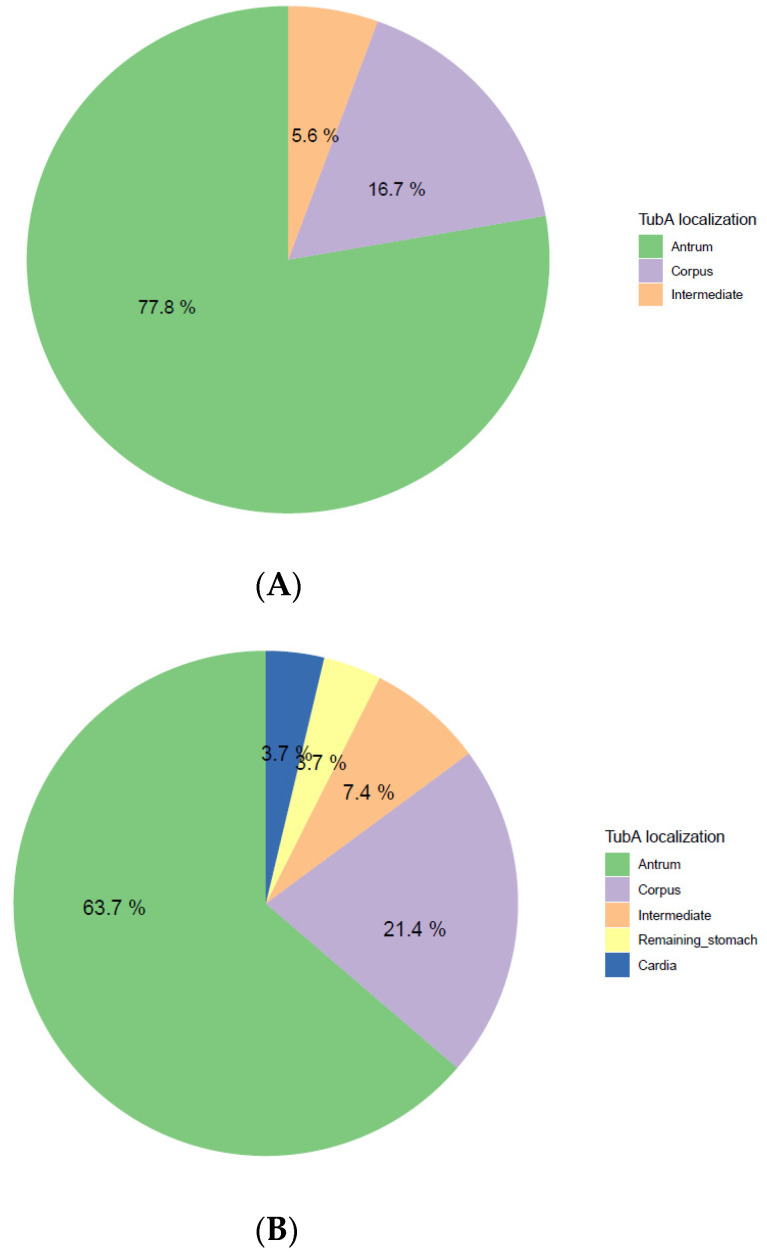
Distribution of intestinal-type adenoma localization within (**A**) Corpus dominant *H. pylori* gastritis and (**B**) *H. pylori* gastritis (**B**). TubA: intestinal-type adenoma.

According to the two-proportion z-test, no significant difference (*p-*value = 0.35) was detected in the main localization of TubAs between Corpus dominant gastritis (77.8% in the antrum) and active *H. pylori*-gastritis (63.7 % in the antrum) (Figure 6).

In lymphocytic gastritis, all adenomas were TubAs, and one of them was associated with FAP.

### 3.6. Association of Gastritis Types with Intestinal Metaplasia and Atrophy with Regard to the OLGIM and OLGA Staging Systems

**Figure 7:**
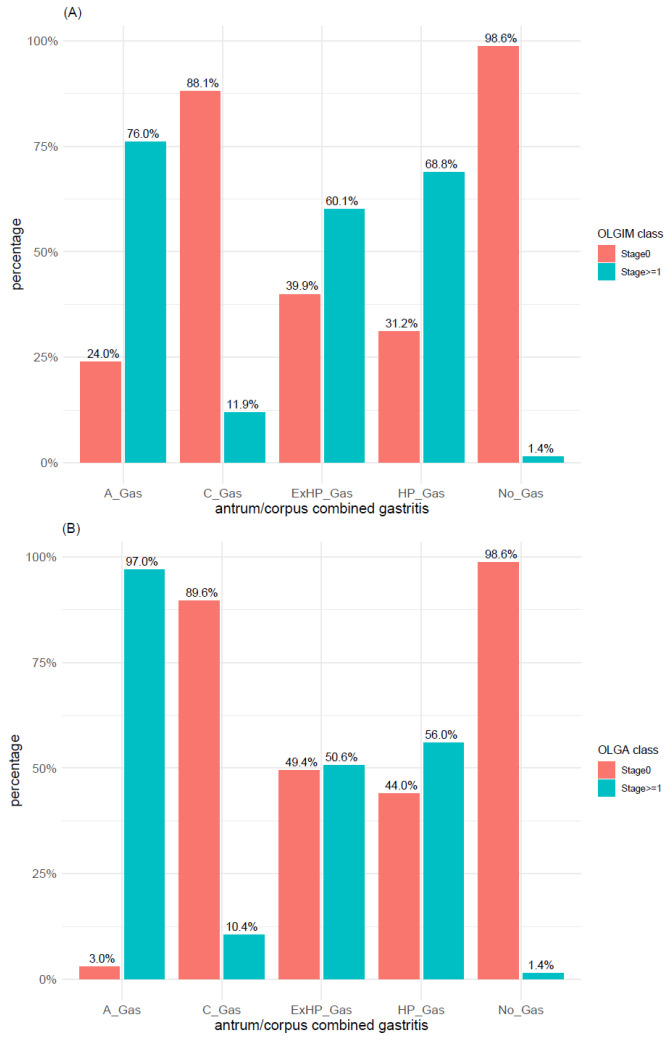
Distribution of (**A**) intestinal metaplasia and (**B**) atrophy within the main gastritis types according to OLGA and OLGIM classes (stage 0 vs. stages 1–4). TubA: intestinal-type adenoma; PGA: pyloric gland adenoma; FovA: foveolar-type adenoma; OGA: oxyntic gland adenoma; A_Gas: autoimmune gastritis; C_Gas: chemical reactive gastritis; ExHP_Gas: Ex-*H. pylori* Gastritis; HP_Gas: *H. pylori* Gastritis; No_Gas: no gastritis; OLGA: operative link on gastritis assessment; OLGIM: operative link on gastric intestinal metaplasia assessment.

Relying on the 2-level classification system described in Materials and Methods (i.e., OLGIM/OLGA stage 0 vs. stage >= 1), we found a significant association between IM/atrophy and the main gastritis types (*p*-values < 0.0001). As expected, a significantly higher number of cases with IM and atrophy was found in A-gastritis, whereas both OLGIM stage 0 and OLGA stage 0 were mainly found in C-gastritis and cases without gastritis (Figure 7A,B).

**Figure 8:**
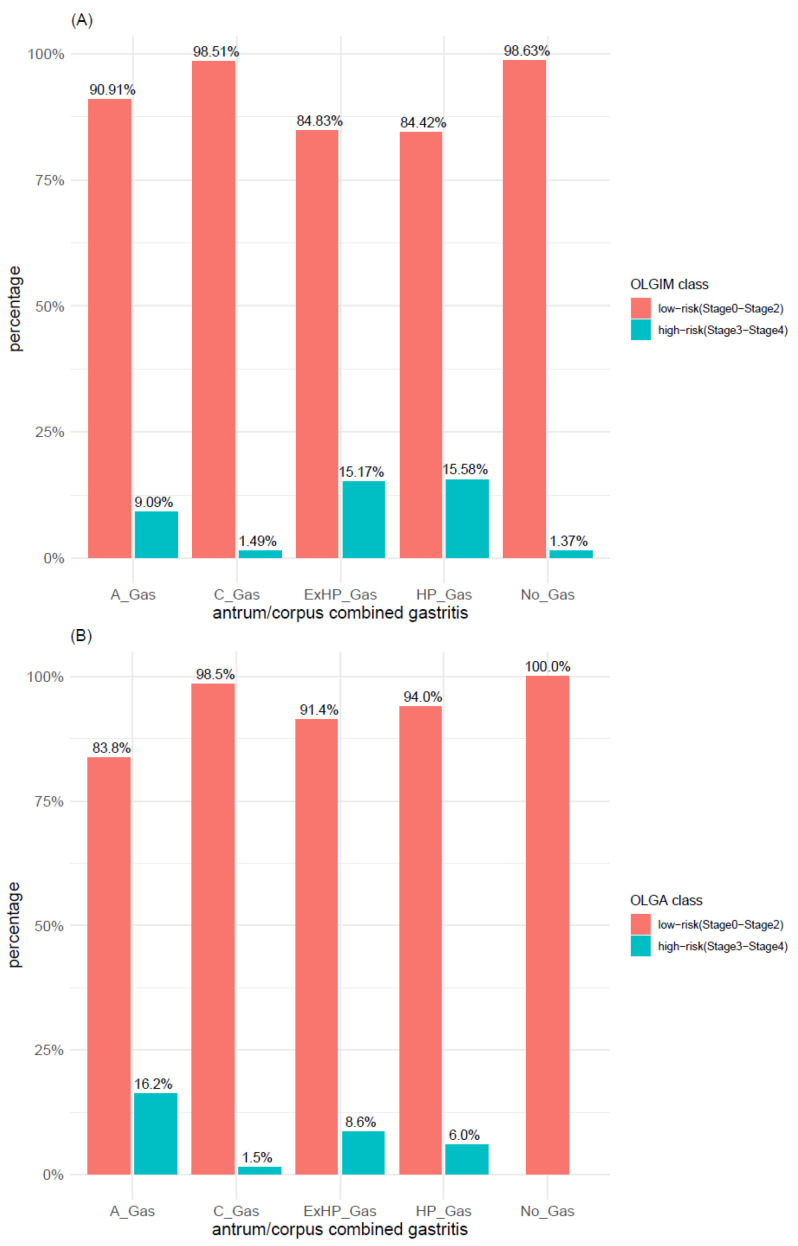
Distribution of (**A**) intestinal metaplasia and (**B**) atrophy within the main gastritis types according to low- and high-risk of OLGA and OLGIM classes. A_Gas: autoimmune gastritis; C_Gas: chemical reactive gastritis; ExHP_Gas: Ex-*H. pylori* Gastritis; HP_Gas: *H. pylori* Gastritis; No_Gas: no gastritis; OLGA: operative link on gastritis assessment; OLGIM: operative link on gastric intestinal metaplasia assessment.

A further analysis performed relying on the low-/high-risk classification revealed that this classification system, depicting a high proportion of low-risk cases within each of the investigated gastritis types, was able to detect the association of A-gastritis with atrophy but not with IM (Figure 8). Ex-*H. pylori*, *H. pylori*, and A-gastritis were the gastritis types characterized by the highest number of cases with high-risk scores for OLGA/OLGIM, whereas, in C-gastritis and “no gastritis”, they accounted for <2%.

**Figure 9:**
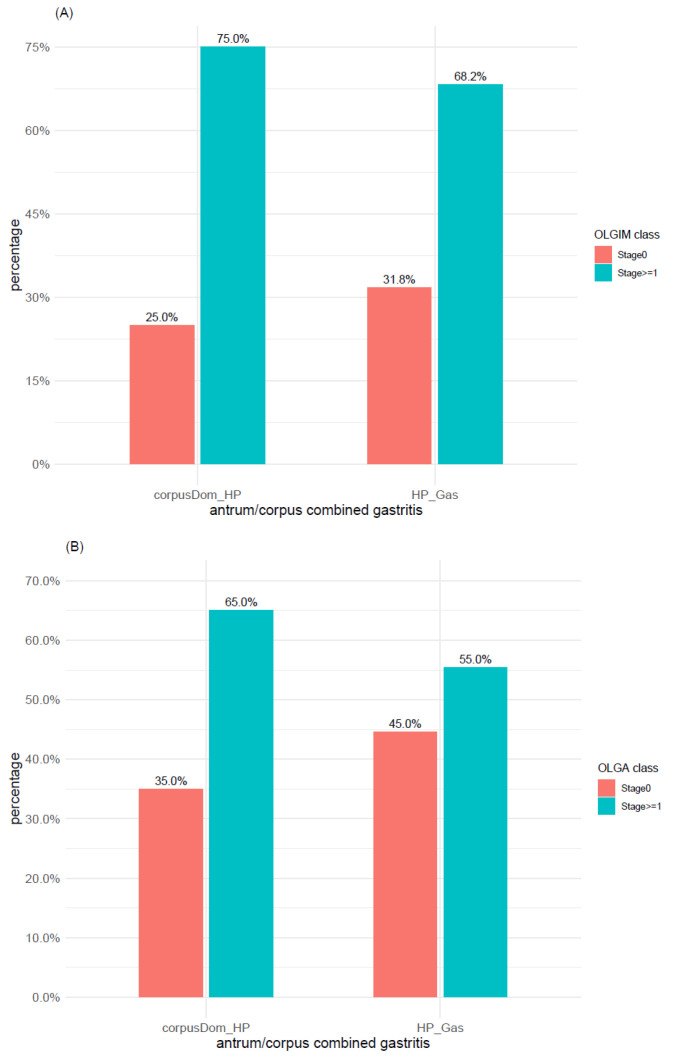
Comparison of *H. pylori* and Corpus dominant *H. pylori* gastritis with regard to (**A**) intestinal metaplasia and (**B**) atrophy. OLGA and OLGIM classes are grouped as stage 0 vs. stages 1–4. corpusDom_HP: Corpus Dominant *H. pylori* Gastritis; HP_Gas: *H. pylori* Gastritis; OLGA: operative link on gastritis assessment; OLGIM: operative link on gastric intestinal metaplasia assessment.

A comparison of Corpus dominant *H. pylori* gastritis versus conventional *H. pylori* gastritis was performed relying on the 2-level classification system OLGIM/OLGA stage 0 vs. stage >= 1. Intestinal metaplasia (IM) was found in 15/20 (75%) cases of Corpus dominant *H. pylori* gastritis and in 144/211 (68.2%) cases of *H. pylori* gastritis (Figure 9A). Atrophy was found in 13/20 (65%) cases of Corpus dominant *H. pylori* gastritis and in 117/212 (55.2%) cases of *H. pylori* gastritis (Figure 9B). No significant differences were found between the two gastritis types regarding OLGIM (*p*-value = 0.71) and OLGA (*p*-value = 0.54) scores.

**Figure 10:**
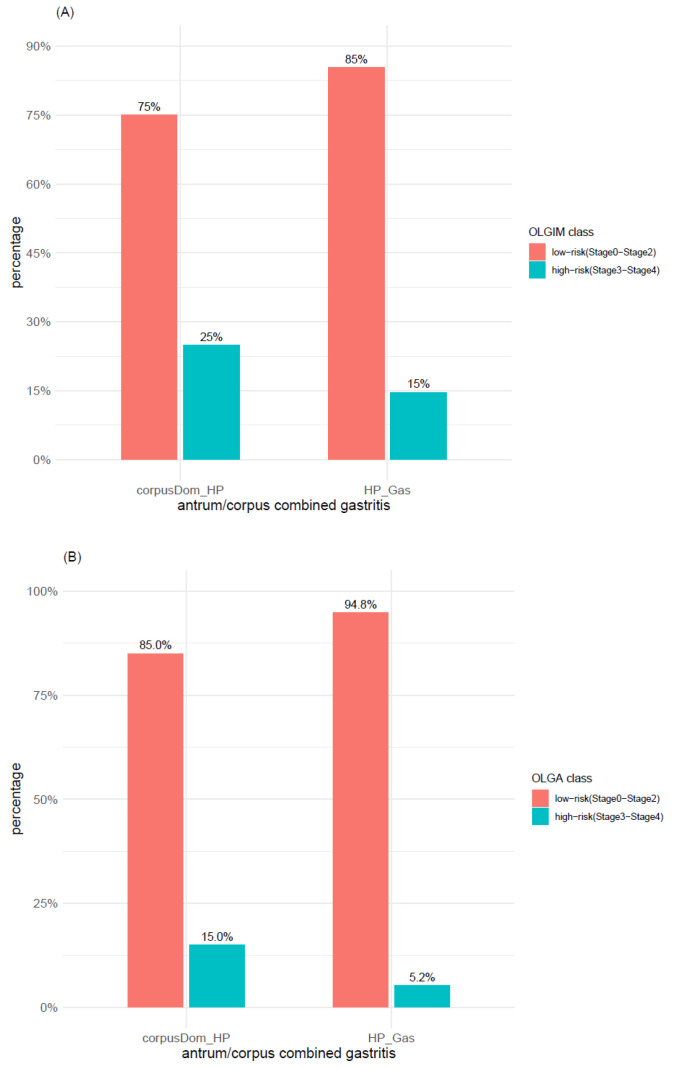
Comparison of *H. pylori* and Corpus dominant *H. pylori* gastritis with regard to (**A**) intestinal metaplasia and (**B**) atrophy. OLGA and OLGIM classes are grouped as low-risk and high-risk. corpusDom_HP: Corpus Dominant *H. pylori* Gastritis; HP_Gas: *H. pylori* Gastritis; OLGA: operative link on gastritis assessment; OLGIM: operative link on gastric intestinal metaplasia assessment.

Comparing Corpus dominant *H. pylori* gastritis versus conventional *H. pylori* gastritis based on the 2-level classification system low-/high-risk, 15/5 (75%) of Corpus dominant *H. pylori* gastritis and 180/211 (85%) of *H. pylori* gastritis cases were grouped as low-risk with OLGIM (Figure 10A), whereas 17/20 (85%) and 201/212 (94.8%), respectively, were grouped as low-risk with OLGA (Figure 10B).

### 3.7. Association of Adenoma Types with Intestinal Metaplasia and Atrophy with Regard to the OLGIM and OLGA Staging Systems

**Figure 11:**
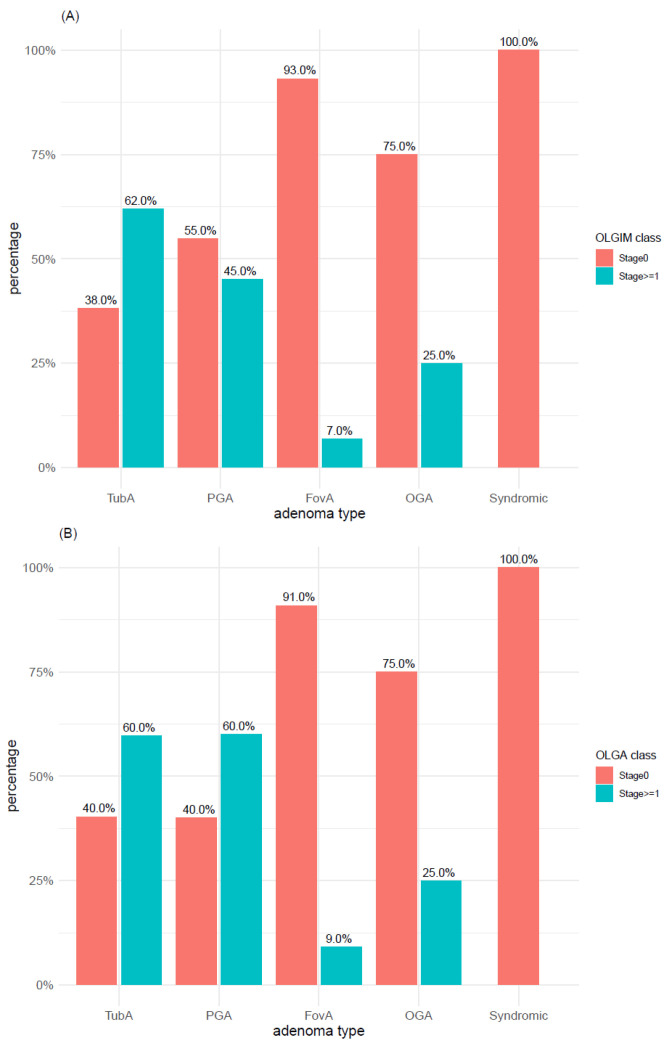
Distribution of (**A**) intestinal metaplasia and (**B**) atrophy within adenoma types according to OLGA and OLGIM classes (stage 0 vs. stages 1–4). TubA: intestinal-type adenoma; PGA: pyloric gland adenoma; FovA: foveolar-type adenoma; OGA: oxyntic gland adenoma; OLGA: operative link on gastritis assessment; OLGIM: operative link on gastric intestinal metaplasia assessment.

**Figure 12:**
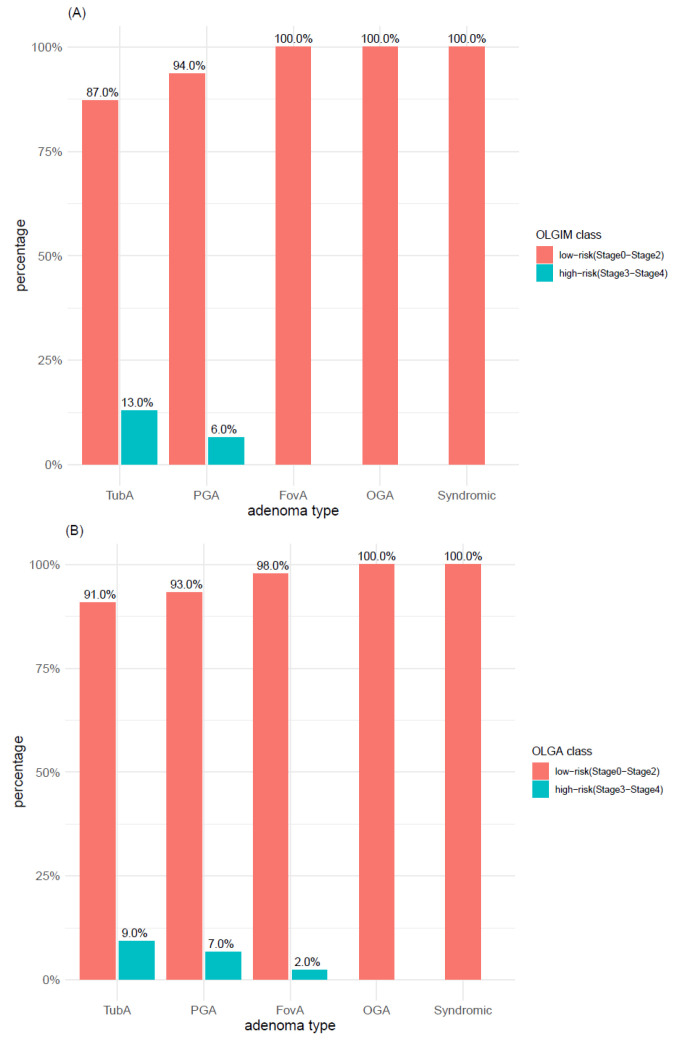
Distribution of (**A**) intestinal metaplasia and (**B**) atrophy within adenoma types according to OLGA and OLGIM classes (low-risk vs. high-risk). TubA: intestinal-type adenoma; PGA: pyloric gland adenoma; FovA: foveolar-type adenoma; OGA: oxyntic gland adenoma.

The distribution of OLGIM and OLGA scores within adenoma types was explored with both 2-level classification systems (i.e., OLGIMA/OLGA stage 0 vs. stage >= 1 and OLGIM/OLGA low-risk vs. high-risk). According to the first 2-level classification system (i.e., OLGIM/OLGA stage 0 vs. stage >= 1), IM (OLGIM stages 1–4) was found in 62% of TubAs and in 45% of PGAs (Figure 11A), whereas atrophy (OLGA stages 1–4) was found in 60% of both adenoma types (Figure 11B). In syndromic cases, neither IM (OLGIM stage >= 1) nor atrophy (OLGA stage >= 1) was found, whereas only a minor proportion of foveolar and oxyntic gland adenomas was associated with IM and atrophy. In TubA, a significant higher proportion of OLGIM (*p*-value < 0.005) and OLGA (*p*-value < 0.01) stage >= 1 was found, compared to stage 0. This difference, however, was lost when applying the low-/high-risk 2-level classification system, which depicted for both IM and atrophy a clear prevalence of low-risk cases within all adenoma types (Figure 12A,B). Adenoma types with the highest number of high-risk cases were TubA and PGA, reaching a percentage of 13% and 6% in OLGIM and 9% and 7% in OLGA.

**Figure 13:**
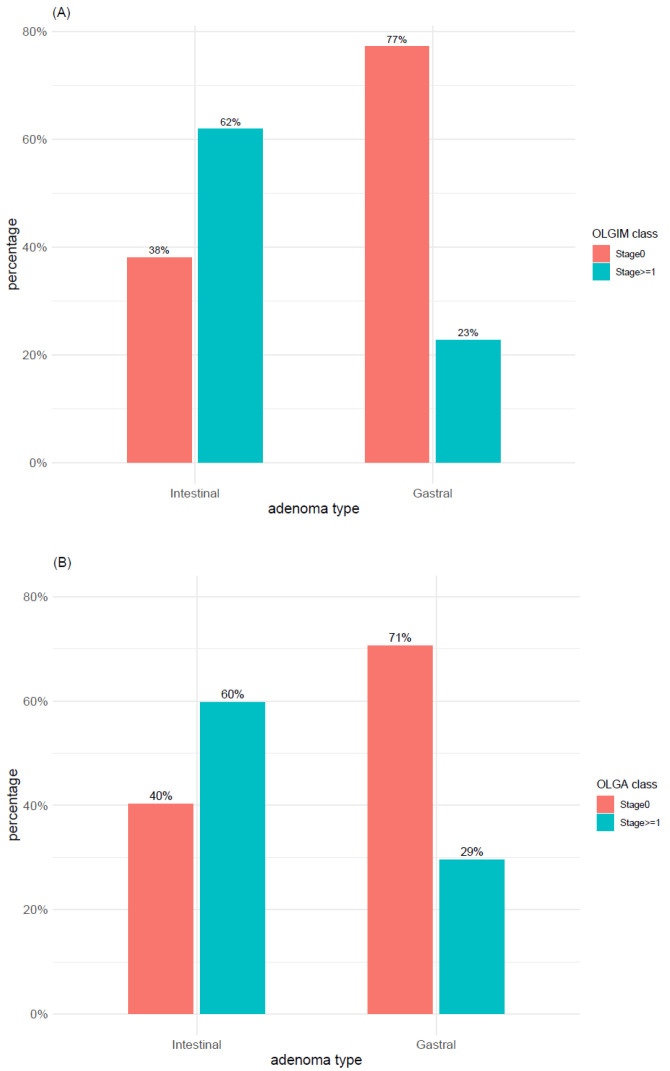
Distribution of (**A**) intestinal metaplasia and (**B**) atrophy within intestinal and gastral adenoma types according to OLGIM and OLGA classes (stage 0 vs. stages 1–4).

The same analyses involving the distribution of OLGIM and OLGA classes were also performed to explore the differences between intestinal-type adenomas and gastric-type adenomas, the latter defined by grouping together FovA, PGA, and OGA cases. A significant association between gastritis types and OLGIM/OLGA classes was detected with a level of significance < 0.001. More specifically, a significantly higher proportion of IM and atrophy (OLGIM/OLGA stages >= 1) was found in intestinal tubular adenoma, whereas a higher number of OLGIM and OLGA stage 0 cases was found in gastric-type adenomas (Figure 13A,B).

**Figure 14:**
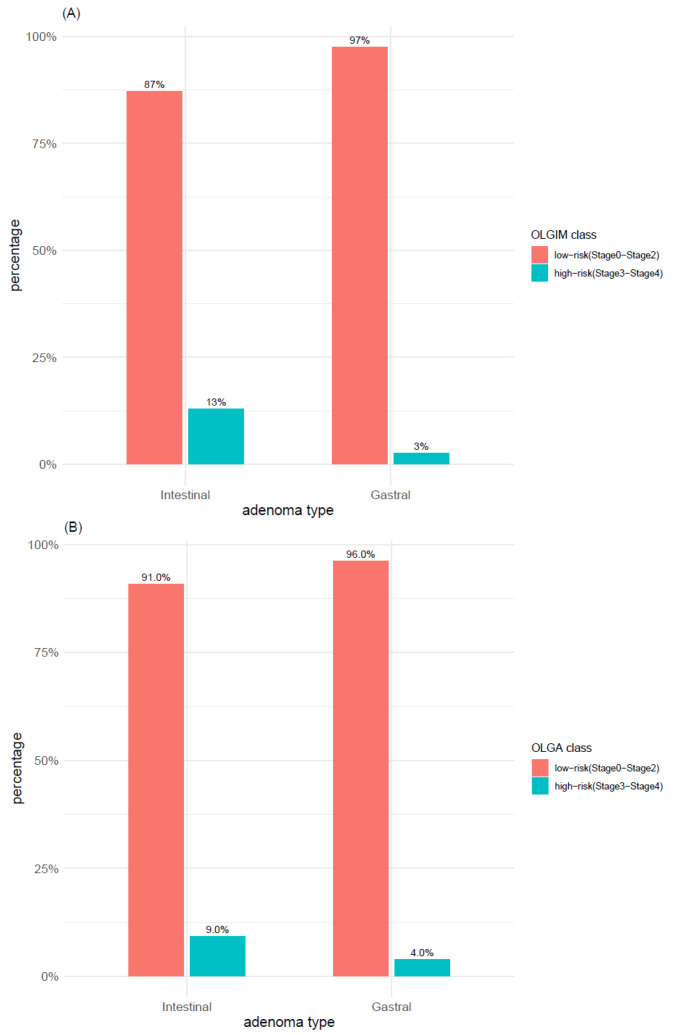
Distribution of (**A**) intestinal metaplasia and (**B**) atrophy within intestinal and gastral adenoma types according to OLGA and OLGIM classes (low-risk vs. high-risk).

When analyzing the distribution of OLGIM and OLGA low-/high-risk classes within intestinal-type and gastric-type adenomas, the differences between the adenoma-types previously detected (Figure 13) seemed to be lost. Indeed, almost all cases belonged to the low-risk group (Figure 14A,B).

## 4. Discussion

We present a consecutive cohort of 1058 gastric adenomas diagnosed between 1987 and 2017 in a single institution with a focus on gastrointestinal pathology.

An increase in the number of diagnoses over the investigated time frame, peaking in 2004, could be detected, probably reflecting the increase in the number of biopsy specimens in recent years. TubA appeared to be the most diagnosed adenoma type throughout the whole period of time. This is in accordance with the literature knowledge describing TubA as the most frequent adenoma type [1]. Diagnoses of gastric-type adenomas were first made in the 1990s, e.g., PGA starting from 1991 and FovA starting from 1999 when first reports on these entities occurred [37,38,39,40]. The systematic diagnosis of both adenoma types starting from 1998 reflects the general acceptance of both lesions as a separate entity dating to the 2000s, going along with publications of larger PGA and FovA cohorts in established journals [15,16,20]. Interestingly, in recent years, the frequency of PGAs seems to be decreasing in favor of FovAs, which may be explained by the constantly expanding use of eradication therapies.

In this cohort, TubA with almost 90% was by far the most common of all adenoma types, followed by FovA (4.3%), PGA (3.4%), syndromic adenoma (2.8%), and OGA (0.4%). The incidence of PGA was slightly higher compared to literature data, which report a frequency of 2.7% [20]. This may be due to the increased awareness of PGA as a distinct entity, and thus an increase in the frequency of PGA diagnoses, after the presentation of the first larger PGA cohort in 2003 [20]. Since our cohort has the consecutive character, it is possible that the figures presented here are more representative than those from selected cohorts.

TubA, both sporadic and syndromic, was the most frequent adenoma type encountered in antral mucosa. However, in Western FAP patients, TubAs are very rare and account for 1% to 2% of gastric adenomas [19], which corresponds to approximately 2.4% syndromic TubAs in our cohort. Among TubAs, the antrum was the predominant localization, whereas for PGA and foveolar gland adenomas, the predominant localization was the corpus, which is consistent with literature results [16,20]. The association between adenoma types and specific mucosal sites has been linked to different types of gastritis and associated changes, which also occur predominantly in respective compartments of the stomach. Since TubA is assumed to arise in gastric mucosa with intestinal metaplasia, it frequently occurs in the context of *H. pylori* gastritis or chronic (autoimmune) gastritis with atrophy [9,16]. Accordingly, in our cohort, sporadic TubA occurred in the background of *H. pylori*, Ex-*H. pylori*, or A-gastritis in 90%, mainly in the antrum (almost 60%). However, more than 60% of syndromic TubAs developed on pristine mucosa almost evenly distributed in the antrum or corpus (53% and 40%, respectively). Our data support an association of TubA with IM by the finding of IM and higher OLGIM scores in *H. pylori* and A-gastritis but also Ex-*H. pylori* gastritis, underlining the character of IM as a persisting mucosal change even after *H. pylori* eradication but probably more a bystander than a real precancerous lesion. Moreover, the frequency of TubA was directly associated with *H. pylori*-, Ex-*H. pylori* and A-gastritis. The rather high frequency of Ex-*H. pylori* gastritis lets us speculate on a point of no return for the development of neoplasia even in absence of active Helicobacter infection, but this discussion needs a much larger cohort and a defined follow-up period. We feel justified to state that our data may fuel the upcoming discussion. There was no significant difference between the frequency of IM and predominant localization of TubA in *H. pylori* and Corpus dominant *H. pylori* gastritis, concluding that the intensity of active inflammation probably has no impact on the development of IM and associated TubAs. Thus, questioning OLGA and OLGIM staging a bit since more than 60% of evaluable cases with adenomas occur in stages 0 and 1 of OLGA and more than 70% in stages 0 and 1 of OLGIM. This leads to the question of whether the benefit of OLGA and OLGIM may be limited to some patients but not being suitable for all individuals (e.g., limiting to *H. pylori* and/or Ex-*H. pylori* cases). This needs to be clarified in future studies with a defined follow-up. There was no significant difference in the distribution of adenoma types of Corpus dominant *H. pylori* gastritis compared to conventional *H. pylori* gastritis. The majority were TubA in both types of gastritis with 18/20 (90.0%) cases compared to 215/219 (98.2%).

In contrast, the predilection of PGA for localization in the corpus could be linked to A-gastritis in almost 60% in our cohort, compared to 36–40% in the literature [18,20]. This difference may be explained by the strong adherence to the guidelines always evaluating the gastritis status with additional antrum and corpus biopsies. PGA is assumed to arise from pyloric gland metaplasia or gastric heterotopia but not IM [41], which explains the absence of a clear association of IM with PGA in our cohort. It also explains the predominant localization in the corpus, since pyloric metaplasia in the context of A-gastritis occurs most frequently in atrophic corpus mucosa [19]. The higher prevalence of PGA in older patients (mean age 74.8 years) and females (in our cohort 72.2%) can also be linked to the association with A-gastritis, which is much more frequent in older females [20]. However, even in the setting of A-gastritis, PGA is said to be a rare lesion [42] and PGA was shown to occur more frequently in the setting of FAP than autoimmune gastritis [19].

According to the WHO classification [1], gastric FovA is exceedingly rare in non-syndromic cases but accounts for about 85% of adenomas in Western FAP patients [19]. This is in contrast to our findings with low numbers of FovA associated with FAP compared to quite high numbers of sporadic FovA. This could be explained by a subset of syndromic cases, which may be hidden within the non-syndromic cases due to lack of clinical information. This could also be the situation in two cases of our cohort with simultaneous FovA and PGA, which is a frequent finding in FAP patients [19]. However, we did not find definite data on the actual frequency of sporadic FovA in the literature, and even in our cohort sporadic FovA accounting for 4.3% of all adenomas was still rare. According to literature, FovA rarely occurs in the setting of longstanding gastritis [1], which is consistent with our data with a background of uninflamed mucosa in almost 60%, and lack of IM (93% OLGIM score 0) or atrophy (91% OLGA score 0) in the majority of cases. This supports the opinion that despite probably several unreported FAP cases, sporadic cases also arise in an uninflamed background.

According to the literature, OGA exclusively arises from an intact oxyntic mucosa that can be found in the cardia, corpus, and fundus [1,30]. Accordingly, in our cohort, OGAs were found in the corpus and cardia only. Since the number of OGA in our cohort was low, we cannot draw any reliable information regarding associations with clinicopathological variables. However, our findings may add information to the current literature, which is based on mainly small OGA cohorts. In four OGA in our cohort, there was no active *H. pylori* gastritis and no predominant gastritis type. IM and simultaneous atrophy occurred in one case with A-gastritis. The findings are consistent with literature data, where a context with *H. pylori*, chronic gastritis, atrophy, or intestinal metaplasia has been largely ruled out [28,31]. Moreover, in our cohort, OGA occurred in male patients only. A male predominance (3:1) has recently been reported in a cohort of 26 OGA [28].

For the analysis of OLGA and OLGIM risk-scores in adenoma types and gastritis types, we applied the two-tiered risk-stratification system (OLGA/OLGIM 0–2 low-risk vs. OLGA/OLGIM 3–4 high-risk) [36] in order to gain information on the risk of progression. The distribution between low-risk and high-risk lesions did not differ significantly between adenoma types, neither when investigating each adenoma type separately, nor when analyzing intestinal-type versus combined gastral-type adenoma. We found similar results in the analysis of OLGA and OLGIM risk-scores in gastritis types, which also showed no significant difference in the number of low- and high-risk cases within all gastritis types. However, in adenomas, the highest number of high-risk cases is found in TubA and PGA, which are the lesions with the highest progression risk among adenoma types [16,20]. Our results point out as well that cases with gastric-type adenoma (PGA, FovA, OGA) in general may not be covered by the risk stratification with OLGIM and OLGA.

Compared to sporadic cases, we found syndromic adenomas in younger patient ages (mean age 42.8 years). This is consistent with reports of gastric adenomas in FAP patients at a mean age of 41.8 years [43]. Younger patient ages in syndromic cases compared to sporadic cases have also been reported in patients with PGA [44,45]. Syndromic adenomas in our cohort were TubAs or FovAs in FAP and TubAs in Gardner syndrome. Wood et al. reported FovAs in 43/50 FAP-associated polyps (84%), PGA in 7/51 (14%), and intestinal-type gastric adenomas in only one FAP patient (2%; fundic gland polyps, hyperplastic polyps, and carcinomas excluded) [19]. However, within the subgroup of syndromic adenomas in our cohort, we found TubAs and FovAs only, with TubA being most frequent with 86.6% of all syndromic adenomas, whereas PGAs were not found. Since the number of syndromic cases in our cohort is quite low, we did not necessarily expect to find PGAs.

The most interesting finding in syndromic cases was the lack of pathologies of the adjacent mucosa in the majority of cases. PGAs of patients with FAP arise in healthy oxyntic mucosa without inflammation or other mucosal damage [9,19]. In our study, syndromic adenomas associated with uninflamed mucosa in both antrum and corpus in 66.7%. Moreover, there was no IM or atrophy in syndromic cases of our cohort, resulting in OLGIM and OLGA stages 0 in all cases. 26.7% of syndromic adenomas occurred in the context of C-gastritis and 3.3% in Ex-*H. pylori* gastritis. Active inflammation was documented in one of 30 cases only. Consequently, in the case of PGA without associated gastritis, the possibility of FAP should be considered. This is, however, different from the results of a Japanese study, reporting *H. pylori* in 58% of FAP patients with gastric adenomas [43]. In this study, the number of gastric adenomas increased with the extent of atrophy in *H. pylori* associated atrophic gastritis [43]. The high prevalence of *H. pylori* in the mentioned study could be explained by the cohort, which was probably of mainly Asian origin and, by the fact, that all types of gastric adenomas were investigated. It is assumed that *H. pylori* is responsible for the higher rate of progression of TubA to carcinoma compared to FovA [46]. However, in the cited study all patients with adenomas had truncating *APC* germline mutations, leading to the conclusion that adenoma development may directly be related to the genetic alteration [43].

## 5. Conclusions

In this study, we present data of 1058 adenomas of different types diagnosed at a single institution during a period of 30 years. To our knowledge, there is no comparable cohort of this size and consecutive character analyzing gastric adenomas and their mucosal environment in detail. However, we cannot yet present clinical follow-up of the patients and we cannot completely exclude that data on a syndromic background are missing in a subset of patients. Our results are mostly consistent with literature data and give insight into the mucosal environment even of rare lesions, e.g., sporadic FovA, which we found in higher frequencies than expected from the literature, and the impact of specific types of gastritis, e.g., corpus dominant gastritis. Moreover, the mucosal environment of quite a high number of syndromic adenomas was analyzed in comparison to non-syndromic cases. Our results support the general assumption of the pathogenesis of syndromic adenomas based on genetic factors rather than gastritis and associated mucosal changes.

## Figures and Tables

**Table 1:** Clinicopathological variables. * percentage with respect to available cases, rounded to the first decimal place; ** familial adenomatous polyposis (FAP)-associated adenomas (intestinal-type Adenoma, foveolar-type adenoma) and Gardner-Syndrome associated intestinal-type adenomas; *** synchronous adenomas; yr: years; NA: not available; OLGA: operative link on gastritis assessment; OLGIM: operative link on gastric intestinal metaplasia assessment. NA: in these cases, there was no gastritis status/OLGIM/OLGA available.

Clinicopathological Variables	*n* (%) *
**Gender**	
Male	570 (53.9)
Female	488 (46.1)
**Type of Adenoma**	
Intestinal-type adenoma	943 (89.1)
Pyloric gland adenoma	36 (3.4)
Foveolar-type adenoma	45 (4.3)
Oxyntic gland adenoma	4 (0.4)
Adenoma associated with hereditary tumor syndromes **	30 (2.8)
**Localization of Adenoma**	
Antrum	575 (54.4)
Corpus	346 (32.7)
Intermediate zone	68 (6.4)
Cardia	39 (3.7)
Remaining stomach	29 (2.7)
Cardia and antrum ***	1 (0.1)
**Type of Gastritis**	
No gastritis	78 (7.8)
*H. pylori* gastritis	214 (21.4)
Corpus dominant *H. pylori* gastritis	20 (2.0)
Ex-*H. pylori* gastritis	359 (36.0)
Chemical reactive gastritis	74 (7.4)
Autoimmune gastritis	248 (24.8)
Lymphocytic gastritis	5 (0.5)
Crohn’s gastritis	1 (0.1)
NA	59
**OLGIM classification**	
0	419 (43.2)
I	319 (32.9)
II	119 (12.3)
III	89 (9.2)
IV	24 (2.5)
NA	88
**OLGA classification**	
0	430 (44.6)
I	175 (18.2)
II	277 (28.7)
III	69 (7.2)
IV	13 (1.3)
NA	94

**Table 2:** Distribution of age at diagnosis within each adenoma type. TubA: intestinal-type adenoma; PGA: pyloric gland adenoma; FovA: foveolar-type adenoma; OGA: oxyntic gland adenoma; SD: standard deviation.

Adenoma Type	Mean (SD)	Median
TubA	71.1 (11.5)	73
PGA	74.8 (8.8)	74.5
FovA	62.4 (13.4)	67
OGA	68.3 (10.1)	70.5
Syndromic	42.8 (13.5)	43

## References

[1] WHO Classification of Tumours Editorial Board (2019). WHO Classification of Tumours. Digestive System Tumours.

[2] Rugge M., Correa P., Dixon M.F., Hattori T., Leandro G., Lewin K., Riddell R.H., Sipponen P., Watanabe H. (2000). Gastric dysplasia: The Padova international classification. Am. J. Surg. Pathol..

[3] Stolte M. (2003). The new Vienna classification of epithelial neoplasia of the gastrointestinal tract: Advantages and disadvantages. Virchows Arch..

[4] Schlemper R.J., Riddell R.H., Kato Y., Borchard F., Cooper H.S., Dawsey S.M., Dixon M.F., Fenoglio-Preiser C.M., Flejou J.F., Geboes K. (2000). The Vienna classification of gastrointestinal epithelial neoplasia. Gut.

[5] Ming S.C. (1979). Dysplasia of gastric epithelium. Front. Gastrointest. Res..

[6] Japanese Gastric Cancer Association (2011). Japanese classification of gastric carcinoma: 3rd English edition. Gastric Cancer.

[7] Carmack S.W., Genta R.M., Schuler C.M., Saboorian M.H. (2009). The current spectrum of gastric polyps: A 1-year national study of over 120,000 patients. Am. J. Gastroenterol..

[8] Shaib Y.H., Rugge M., Graham D.Y., Genta R.M. (2013). Management of gastric polyps: An endoscopy-based approach. Clin. Gastroenterol. Hepatol..

[9] Brosens L.A., Wood L.D., Offerhaus G.J., Arnold C.A., Lam-Himlin D., Giardiello F.M., Montgomery E.A. (2016). Pathology and Genetics of Syndromic Gastric Polyps. Int. J. Surg. Pathol..

[10] Pimentel-Nunes P., Dinis-Ribeiro M., Ponchon T., Repici A., Vieth M., De Ceglie A., Amato A., Berr F., Bhandari P., Bialek A. (2015). Endoscopic submucosal dissection: European Society of Gastrointestinal Endoscopy (ESGE) Guideline. Endoscopy.

[11] Rugge M., Cassaro M., Di Mario F., Leo G., Leandro G., Russo V.M., Pennelli G., Farinati F., Interdisciplinary Group on Gastric Epithelial D. (2003). The long term outcome of gastric non-invasive neoplasia. Gut.

[12] Li D., Bautista M.C., Jiang S.F., Daryani P., Brackett M., Armstrong M.A., Hung Y.Y., Postlethwaite D., Ladabaum U. (2016). Risks and Predictors of Gastric Adenocarcinoma in Patients with Gastric Intestinal Metaplasia and Dysplasia: A Population-Based Study. Am. J. Gastroenterol..

[13] Tomasulo J. (1971). Gastric polyps. Histologic types and their relationship to gastric carcinoma. Cancer.

[14] Kim Y.J., Park J.C., Kim J.H., Shin S.K., Lee S.K., Lee Y.C., Chung J.B. (2010). Histologic diagnosis based on forceps biopsy is not adequate for determining endoscopic treatment of gastric adenomatous lesions. Endoscopy.

[15] Abraham S.C., Park S.J., Lee J.H., Mugartegui L., Wu T.T. (2003). Genetic alterations in gastric adenomas of intestinal and foveolar phenotypes. Mod. Pathol..

[16] Abraham S.C., Montgomery E.A., Singh V.K., Yardley J.H., Wu T.T. (2002). Gastric adenomas: Intestinal-type and gastric-type adenomas differ in the risk of adenocarcinoma and presence of background mucosal pathology. Am. J. Surg. Pathol..

[17] Park D.Y., Srivastava A., Kim G.H., Mino-Kenudson M., Deshpande V., Zukerberg L.R., Song G.A., Lauwers G.Y. (2008). Adenomatous and foveolar gastric dysplasia: Distinct patterns of mucin expression and background intestinal metaplasia. Am. J. Surg. Pathol..

[18] Chen Z.M., Scudiere J.R., Abraham S.C., Montgomery E. (2009). Pyloric gland adenoma: An entity distinct from gastric foveolar type adenoma. Am. J. Surg. Pathol..

[19] Wood L.D., Salaria S.N., Cruise M.W., Giardiello F.M., Montgomery E.A. (2014). Upper GI tract lesions in familial adenomatous polyposis (FAP): Enrichment of pyloric gland adenomas and other gastric and duodenal neoplasms. Am. J. Surg. Pathol..

[20] Vieth M., Kushima R., Borchard F., Stolte M. (2003). Pyloric gland adenoma: A clinico-pathological analysis of 90 cases. Virchows Arch..

[21] Kushima R., Ruthlein H.J., Stolte M., Bamba M., Hattori T., Borchard F. (1999). ‘Pyloric gland-type adenoma’ arising in heterotopic gastric mucosa of the duodenum, with dysplastic progression of the gastric type. Virchows Arch..

[22] Takei K., Watanabe H., Itoi T., Saito T. (1996). p53 and Ki-67 immunoreactivity and nuclear morphometry of ‘carcinoma-in-adenoma’ and adenoma of the gall-bladder. Pathol. Int..

[23] Matsubara A., Sekine S., Kushima R., Ogawa R., Taniguchi H., Tsuda H., Kanai Y. (2013). Frequent *GNAS* and *KRAS* mutations in pyloric gland adenoma of the stomach and duodenum. J. Pathol..

[24] Kushima R., Vieth M., Borchard F., Stolte M., Mukaisho K., Hattori T. (2006). Gastric-type well-differentiated adenocarcinoma and pyloric gland adenoma of the stomach. Gastric Cancer.

[25] Lee S.E., Kang S.Y., Cho J., Lee B., Chang D.K., Woo H., Kim J.W., Park H.Y., Do I.G., Kim Y.E. (2014). Pyloric gland adenoma in Lynch syndrome. Am. J. Surg. Pathol..

[26] Vieth M., Kushima R., Mukaisho K., Sakai R., Kasami T., Hattori T. (2010). Immunohistochemical analysis of pyloric gland adenomas using a series of Mucin 2, Mucin 5AC, Mucin 6, CD10, Ki67 and p53. Virchows Arch..

[27] Ma C., Giardiello F.M., Montgomery E.A. (2014). Upper tract juvenile polyps in juvenile polyposis patients: Dysplasia and malignancy are associated with foveolar, intestinal, and pyloric differentiation. Am. J. Surg. Pathol..

[28] Ushiku T., Kunita A., Kuroda R., Shinozaki-Ushiku A., Yamazawa S., Tsuji Y., Fujishiro M., Fukayama M. (2020). Oxyntic gland neoplasm of the stomach: Expanding the spectrum and proposal of terminology. Mod. Pathol..

[29] Chan K., Brown I.S., Kyle T., Lauwers G.Y., Kumarasinghe M.P. (2016). Chief cell-predominant gastric polyps: A series of 12 cases with literature review. Histopathology.

[30] Singhi A.D., Lazenby A.J., Montgomery E.A. (2012). Gastric adenocarcinoma with chief cell differentiation: A proposal for reclassification as oxyntic gland polyp/adenoma. Am. J. Surg. Pathol..

[31] Ueyama H., Matsumoto K., Nagahara A., Hayashi T., Yao T., Watanabe S. (2014). Gastric adenocarcinoma of the fundic gland type (chief cell predominant type). Endoscopy.

[32] Wu T.-T., Hamilton S.R. (1999). Lymphocytic Gastritis: Association with Etiology and Topology. Am. J. Surg. Pathol..

[33] Rugge M., Correa P., Di Mario F., El-Omar E., Fiocca R., Geboes K., Genta R.M., Graham D.Y., Hattori T., Malfertheiner P. (2008). OLGA staging for gastritis: A tutorial. Dig. Liver Dis..

[34] Capelle L.G., de Vries A.C., Haringsma J., Ter Borg F., de Vries R.A., Bruno M.J., van Dekken H., Meijer J., van Grieken N.C., Kuipers E.J. (2010). The staging of gastritis with the OLGA system by using intestinal metaplasia as an accurate alternative for atrophic gastritis. Gastrointest. Endosc..

[35] Rugge M., Genta R.M. (2005). Staging and grading of chronic gastritis. Hum. Pathol..

[36] Rugge M., Genta R.M., Graham D.Y., Di Mario F., Vaz Coelho L.G., Kim N., Malfertheiner P., Sugano K., Tsukanov V., Correa P. (2016). Chronicles of a cancer foretold: 35 years of gastric cancer risk assessment. Gut.

[37] Hattori T. (1985). Morphological range of hyperplastic polyps and carcinomas arising in hyperplastic polyps of the stomach. J. Clin. Pathol..

[38] Kushima R., Muller W., Stolte M., Borchard F. (1996). Differential p53 protein expression in stomach adenomas of gastric and intestinal phenotypes: Possible sequences of *p53* alteration in stomach carcinogenesis. Virchows Arch..

[39] Rubio C.A., Kato Y., Kitagawa T., Sugano H., Grimelius L. (1988). Intramucosal cysts of the stomach. VIII: Histochemical studies. APMIS.

[40] Borchard F., Ghanei A., Koldovsky U., Hengels K.J., Bückmann F.W. (1990). Gastrale Differenzierung in Adenomen der Magenschleimhaut. Immunhistochemische und elektronenmikroskopische Untersuchungen. Verh. Dtsch. Ges. Pathol..

[41] Vieth M., Montgomery E.A. (2014). Some observations on pyloric gland adenoma: An uncommon and long ignored entity!. J. Clin. Pathol..

[42] Park J.Y., Cornish T.C., Lam-Himlin D., Shi C., Montgomery E. (2010). Gastric lesions in patients with autoimmune metaplastic atrophic gastritis (AMAG) in a tertiary care setting. Am. J. Surg. Pathol..

[43] Nakamura S., Matsumoto T., Kobori Y., Iida M. (2002). Impact of *Helicobacter pylori* infection and mucosal atrophy on gastric lesions in patients with familial adenomatous polyposis. Gut.

[44] Hackeng W.M., Montgomery E.A., Giardiello F.M., Singhi A.D., Debeljak M., Eshleman J.R., Vieth M., Offerhaus G.J., Wood L.D., Brosens L.A. (2017). Morphology and genetics of pyloric gland adenomas in familial adenomatous polyposis. Histopathology.

[45] Hashimoto T., Ogawa R., Matsubara A., Taniguchi H., Sugano K., Ushiama M., Yoshida T., Kanai Y., Sekine S. (2015). Familial adenomatous polyposis-associated and sporadic pyloric gland adenomas of the upper gastrointestinal tract share common genetic features. Histopathology.

[46] Park J.G., Park K.J., Ahn Y.O., Song I.S., Choi K.W., Moon H.Y., Choo S.Y., Kim J.P. (1992). Risk of gastric cancer among Korean familial adenomatous polyposis patients. Report of three cases. Dis. Colon Rectum.

